# Pathophysiological Mechanisms of Cognitive Impairment and Neurodegeneration by *Toxoplasma gondii* Infection

**DOI:** 10.3390/brainsci10060369

**Published:** 2020-06-12

**Authors:** Gloria Ortiz-Guerrero, Rodrigo E. Gonzalez-Reyes, Alejandra de-la-Torre, German Medina-Rincón, Mauricio O. Nava-Mesa

**Affiliations:** 1 Department of Neurology, University of Kansas Medical Center, Kansas City, KS 66160, USA; gloriaortiz9024@gmail.com; 2 GI en Neurociencias-NeURos, Escuela de Medicina y Ciencias de la Salud, Universidad del Rosario, Bogotá 111221, Colombia; rodrigo.gonzalez@urosario.edu.co (R.E.G.-R.); alejadelatorre@yahoo.com (A.d.-l.-T.); germanj.medina@urosario.edu.co (G.M.-R.)

**Keywords:** *Toxoplasma gondii*, Alzheimer’s disease, amyloid-beta, dementia, cognitive impairment

## Abstract

*Toxoplasma gondii* is an obligate intracellular parasite considered one of the most successful pathogens in the world, owing to its ability to produce long-lasting infections and to persist in the central nervous system (CNS) in most warm-blooded animals, including humans. This parasite has a preference to invade neurons and affect the functioning of glial cells. This could lead to neurological and behavioral changes associated with cognitive impairment. Although several studies in humans and animal models have reported controversial results about the relationship between toxoplasmosis and the onset of dementia as a causal factor, two recent meta-analyses have shown a relative association with Alzheimer’s disease (AD). AD is characterized by amyloid-β (Aβ) peptide accumulation, neurofibrillary tangles, and neuroinflammation. Different authors have found that toxoplasmosis may affect Aβ production in brain areas linked with memory functioning, and can induce a central immune response and neurotransmitter imbalance, which in turn, affect the nervous system microenvironment. In contrast, other studies have revealed a reduction of Aβ plaques and hyperphosphorylated tau protein formation in animal models, which might cause some protective effects. The aim of this article is to summarize and review the newest data in regard to different pathophysiological mechanisms of cerebral toxoplasmosis and their relationship with the development of AD and cognitive impairment. All these associations should be investigated further through clinical and experimental studies.

## 1. Introduction

Cognitive deficits are prevalent in the elderly population, with a broad spectrum of neurological disorders, ranging from mild cognitive impairment (MCI) to severe forms such as dementia. Alzheimer’s disease (AD) is the most common type of dementia, and is a neurodegenerative condition characterized by memory loss and the weakening of other neurological functions such as orientation, language, executive function, sensory perception, and attention [1,2]. It accounts for two-thirds of all dementia cases and affects 7% of people older than 65 years and 40% of people older than 80 years [3]. Besides neurodegeneration, extracellular deposition of amyloid-β (Aβ) plaques and the accumulation of neurofibrillary tangles (NFTs) of hyperphosphorylated tau protein are also considered histopathologic hallmarks of AD [4]. Idiopathic AD has several risks and associated factors, including genetic (i.e., apolipoprotein E (APOE) ε4), environmental, and lifestyle conditions such as sedentarism, dietary factors, cognitive activity, polypharmacy and chronic stress, among others [5,6,7]. Research evidence has suggested an association between infectious pathogens such as *Chlamydia pneumonia*, *Helicobacter pylori*, *Borrelia burgdorferi*, spirochetes, cytomegalovirus (CMV) and herpes simplex virus (HSV) type I, in the appearance of late-onset AD [8,9]. In recent years, another pathogen, *Toxoplasma gondii*, has been proposed as a risk factor in the pathophysiology of AD, as well as in other neuropsychiatric disorders, such as schizophrenia [10], bipolar disorder type I [11], migraine [12], and obsessive-compulsive disorder [13]. To date, several studies have shown a correlation between toxoplasmosis and dementia in both animal and human models. For instance, Kusbeci et al. [14] found higher anti-*Toxoplasma gondii* IgG antibodies levels among AD patients compared with a control group. Additionally, animal models have found that *T. gondii* infections may induce the histopathological hallmarks of AD such as Aβ plaques and hyperphosphorylated tau in the hippocampus and prefrontal cortex [15]. In addition, animals infected with *T. gondii* that were exposed to subdoses of Aβ_1–42_ showed an increase in cognitive impairment [16]. Nevertheless, other groups have found no involvement or even favorable effects of the immunomodulation induced by *T. gondii* on AD. For instance, McGovern et al. [17] found that chronic parasite infection in mice had no impact on age-associated decline in cognitive functions and Jung et al. [18] establish a decrease in Aβ plaque formation in a *Toxoplasma* positive murine model. Moreover, in clinical studies, Perry et al. [19] reported no differences in anti-*Toxoplasma* IgG antibodies levels between AD patients and control groups. Similarly, in a recent case-control study (n = 344 patients), *Toxoplasma* infection (assessed by anti-*T. gondii* IgM and IgG antibodies) and neurological disorders were not related [20], and neither was it associated with dementia in older adults in Africa [21]. However, in two recent meta-analyses, a relative association between *Toxoplasma* infection and AD was found [22,23]. Considering the clinical relationship between toxoplasmosis, cognitive impairment and neurodegenerative disorders, the aim of this article is to review the pathological effects of *Toxoplasma gondii* infection in the nervous system and discuss its role in the pathophysiology of AD and cognitive impairment from a neurobiological perspective. 

## 2. Toxoplasma Gondii Effects in the CNS

### 2.1. Parasite Transmission and Dissemination to the Brain

Although some authors suggest that there is a lack of tropism of *T. gondii* towards specific functional systems in the brain, several preclinical studies have found that *Toxoplasma* has a preference for particular brain areas such as the amygdala, frontal cortex, association cortices and hippocampus [24,25]. Correspondingly, molecular and functional neuroimaging studies suggest that chronic toxoplasmosis may affect neuronal connectivity in the somatosensory cortex and synaptic protein composition in the neocortex, hippocampus, and subcortical areas [26,27]. Notably, some of these brain regions are associated with memory impairment, neuropsychiatric symptoms, and disorientation during AD progress. In contrast, low levels of tissue encysted with parasites or brain lesions have been reported in the cerebellum, brainstem, and myelinated axons, among others [24]. 

*T. gondii*, an apicomplexan and intracellular parasite, and possibly the most successful parasite worldwide [28], has infected approximately one-third of the world’s population [29]. This parasite can infect any nucleated cell in warm-blooded animals, including humans and birds, which are some of the intermediate hosts [30]. In these hosts, *T. gondii* exists in two interchangeable stages: tachyzoite, which is the active and lytic form of the parasite and may cause life-threatening diseases, and bradyzoite, which is the encysted and slow-growing form, capable of building cysts mostly in the brain and muscle tissues [29,31]. The infection is frequently acquired by the digestion of tissue cysts in undercooked meat or by direct contact of highly infective oocysts shed in feces by felines [32], which are also found in water sources and food supplies [33]. Other less common transmissions are organ transplants [34] and vertical infection during pregnancy [31]. Therefore, some sociodemographic and environmental factors, as well as the immune state of the host are related to the transmission, dissemination, and infection of this parasite. 

After the digestion of tissue cysts or oocysts, *T. gondii* reaches the stomach and travels to the gut where it infects enterocytes. This triggers the recruitment and activation of innate immune cells, including monocytes and dendritic cells, which in turn, are infected by the parasite [35]. *T. gondii* induces a hypermigratory state of dendritic cells and inflammatory monocytes, which leads to alterations of the host cells’ actin cytoskeleton, upregulation of the CCR7 chemokine receptor, and activation of gamma-aminobutyric acid (GABA) receptor signaling [29,36,37].

The access of pathogens, cells, and proteins from the blood into the brain is more complicated than the access from the blood into other tissues, due to the presence of the impermeable blood-brain barrier (BBB) [38]. The BBB is composed of endothelial cells with a high number of tight junctions that are supported by a basement membrane. In addition, pericytes and astrocytic endfeet surround the endothelial cells, providing structural and biochemical support while also preventing materials from crossing the endothelium and entering the brain parenchyma [29]. 

**Figure 1:**
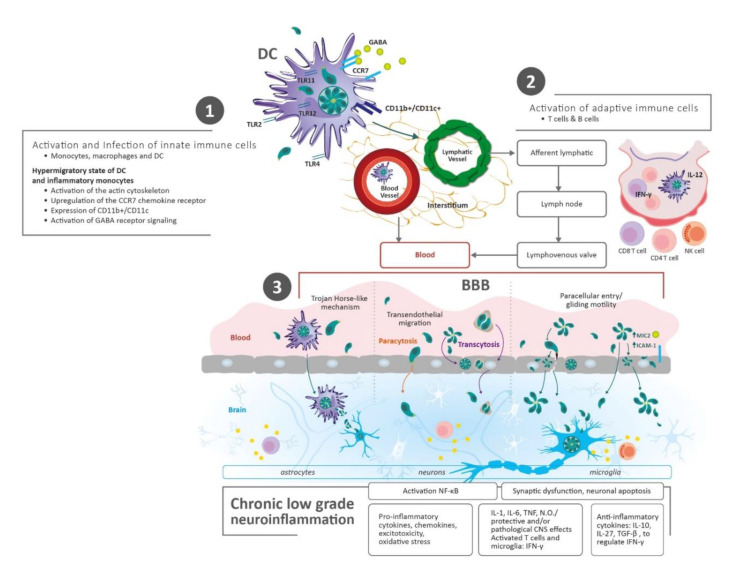
Parasite Transmission and Dissemination to the Brain. (**1**) After human infection with *Toxoplasma gondii* via the oral route (tissue cysts and oocysts), early immune events start in the gut tissue of the host. Innate immune cells are infected and activated, simultaneously. These cells migrate rapidly and spread hematogenously, reaching lymph nodes and peripheral tissues, such as the brain. (**2**) Adaptive immune cells (T and B cells) are stimulated by antigen presenting cells (i.e., dendritic cells—DC) in lymph nodes. In the same way, these inflammatory cells migrate and spread through the blood, disrupting the blood–brain barrier (BBB) and getting into the brain. (**3**) Mechanisms for the transfer of the parasite from the blood to the brain: I—Through infected immune cells: DC diapedesis/Trojan horse-like mechanism. Leukocytes and DC cross the BBB through the endothelial cells or via modifying tight junctions; II—Direct entry of tachyzoites/penetration of the BBB: (a) Paracytosis (through intracellular junctions), (b) Transcytosis (transportation through vesicles), (c) Paracellular entry (increase in the parasite’s microneme protein 2 (MIC2) expression/interaction with the host cell’s intercellular adhesion molecule 1 (ICAM-1), and gliding motility). Abbreviations: Blood–brain barrier (BBB); C-C chemokine receptor type 7 (CCR7); Cluster of differentiation (CD); Central Nervous System (CNS); Dendritic Cells (DC); γ-aminobutyric acid (GABA); Intercellular adhesion molecule 1 (ICAM-1); Interleukin (IL); Interferon gamma (IFN-γ); Monocyte chemoattractant protein-1 (MCP-1); Microneme protein 2 (MIC2); Natural Killer (NK); Nitric oxide (N.O); Nuclear factor kappa-light-chain-enhancer of activated B cells (NF-κB); Toll-like receptor (TLR). Transforming growth factor-beta (TGF-β); Tumor Necrosis Factor (TNF).

Several mechanisms have been proposed for the transfer of the parasite from the blood to the brain: (a) Trojan Horse-like mechanism in which monocytes and other myeloid-derived cells, infected by *Toxoplasma gondii*, could extravasate from capillaries into the brain; (b) Transendothelial migration across human endothelial cells, which is dependent on the attachment of the parasite to CD11b/ICAM1 integrins expressed by the infected cells, and occurs regularly under fluidic shear stress conditions [29,39,40]; (c) Paracellular entry of the parasite into the CNS through actin-myosin motors, displaying a movement mechanism termed “gliding motility”. This movement is thought to aid *T. gondii* in the evasion of tight and paracellular junctions, immune barriers, and polarized cell monolayers, including the BBB. Barragan et al. [41] found an increase in the parasite’s adhesive microneme protein 2 (MIC2) expression followed by the interaction with the host cell’s intercellular adhesion molecule 1 (ICAM-1). This upregulation thereby allows the movement of the parasite through non-permissive biological barriers [42] (Figure 1). 

Once monocytes and dendritic cells are infected and activated, simultaneously, Th1 cells are stimulated as part of the adaptive immune response. As previously mentioned, all these inflammatory cells become excitable, start to migrate rapidly and spread hematogenously, reaching peripheral tissues, such as the brain, and disrupting the BBB [43]. This was proven through a mouse model, where *T. gondii* infected cells expressing CD11b+/CD11c+ integrins were found in the brain-extravascular space [39], and by determining changes in transcriptional regulation profiles in brain endothelial cells. Cells expressing CD11b, either with or without CD11c, are likely candidate cells for the intracellular transport of *T. gondii* across the BBB [44]. On the other hand, some tachyzoites that have escaped from the immune response reach the brain by transcytosis (transfer of an extracellular macromolecule from one side of a cell to the other via pinocytic-derived membrane bound vesicles) or paracytosis (crossing of the endothelial barrier through intracellular junctions) [29]. The models which support the above statement were demonstrated by transwell tissue culture systems and two-photon in vivo imaging, where tachyzoites were able to cross the retinal vascular endothelial cell layers and BBB, respectively [45,46]. 

### 2.2. Chronic Immune Response in the Brain

Chronic low-grade inflammation has a significant role in the pathogenesis of AD. Several studies have shown that toll-like receptors (TLR) contribute to neuroinflammation during AD progression [47,48]. Correspondingly, chronic toxoplasmic encephalitis in mice increases the levels of TLR11 (a specific receptor for *T. gondii*) in neurons, astrocytes, and microglia [49]. In addition, *Toxoplasma* infection activates the nuclear factor kappa B (NF-κB) pathway with the subsequent activation of astrocytes and microglia, synaptic dysfunction, and neuronal apoptosis [50]. Aβ also activates NF-κB signaling in astrocytes [51]. NF-κB is responsible for the release of pro-inflammatory cytokines and chemokines, excitotoxicity and oxidative stress in several models of AD [52]. 

After *T. gondii* reaches the brain, activated CD4+ and CD8+ T cells, as well as influxes of macrophages and natural killer (NK) cells, seem to control *Toxoplasma* infection. Afterward, there is an increase in the production of cytokines and mediators such as interleukin 1 (IL-1), IL-6, tumor necrosis factor (TNF), and nitric oxide, causing protective and/or pathological CNS effects [53,54]. Besides, activated T cells and microglia produce interferon-gamma (IFN-γ), which helps to control the parasite replication by triggering degradation of parasitophorous vacuoles, enhancing antigen presentation and major histocompatibility complex (MHC) genes, and upregulating antiparasitic factors such as nitric oxide synthetase and indoleamine dioxygenase [55]. Moreover, anti-inflammatory cytokines such as IL-10 and IL-27 are produced to regulate IFN-γ and other proinflammatory cytokines [56].

**Figure 2:**
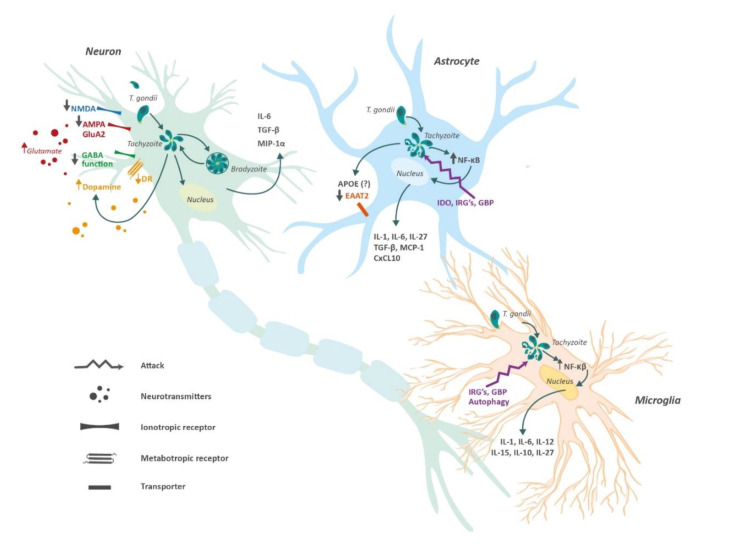
Central nervous system cells and *Toxoplasma gondii*. Tachyzoites from *T. gondii* can enter astrocytes, microglia and neurons. The presence of *T. gondii* induces functional changes in these cells which promote the release of anti- and pro-inflammatory cytokines, and alter both gliotransmission and neurotransmission. Neurons lack an effective defense system against the parasite, therefore, cysts with bradyzoites can form perpetuating the infection. In contrast, astrocytes and microglia possess different mechanisms to protect against the presence of tachyzoites. Abbreviations: α-amino-3-hydroxy-5-methyl-4-isoxazolepropionic acid receptor (AMPA); Apolipoprotein E (APOE); C-X-C motif chemokine 10 (CXCL10); excitatory amino acid transporter (EAAT); γ-aminobutyric acid (GABA); guanylate-binding protein (GBP); immunity-related GTPases (IRG´s); indoleamine 2,3-dioxygenase (INO); Interleukin (IL); monocyte chemoattractant protein 1 (MCP-1); Macrophage Inflammatory Proteins 1 alpha (MIP-1α); nuclear factor kappa-light-chain-enhancer of activated B cells (NF-κB); transforming growth factor beta (TGF-β).

Tachyzoites can infect astrocytes, neurons, and microglia cells, with neurons being the ones predominantly infected by the parasite since they provide unique metabolic and immunological features for bradyzoite development [57]. For instance, neurons cannot respond to stimulation against TNF or INF-γ, hence proving unable to build an appropriate antiparasitic response [58]. Furthermore, in vitro studies have demonstrated that microglia and astrocytes are capable of inhibiting parasite replication via nitric oxide mechanism [59]. These bradyzoites are able to form cysts within a glycoprotein-rich wall. Cysts help *T. gondii* to maintain structural and nutrient needs while evading immune-mediated destruction [60]. Another mechanism for controlling *Toxoplasma* infection involves intracerebral T cells (CD4+ and CD8+). These cells interact with microglia and astrocytes in order to prevent neuronal damage [61]. A fine-tuned balance between astrocytes and microglia is necessary for adequate immunological modulation. In fact, astrocytic transforming growth factor beta (TGF-β) is essential during toxoplasmic infection to reduce neuronal inflammation and brain injury [62] (Figure 2). 

## 3. Amyloid Beta Plaques Accumulation and Tau Pathology

One of the hallmarks in the pathophysiology of AD is the accumulation of Aβ plaques leading to oxidative and inflammatory damage in the brain. These Aβ plaques come from the cleavage of amyloid precursor proteins (APP) by β and γ secretases [6,63]. Curiously, two studies have proven that chronic toxoplasmosis in mice can represent a protective factor for cognitive impairment by showing a decrease in Aβ plaque formation [18,64]. For instance, Jung et al. [18] found that the protection conferred by *T. gondii* was induced by anti-inflammatory cytokines, such as TGF-β and IL-10, while Möhle et al. [64] suggested that the protection was owed to infiltration of monocytes capable of phagocytosing Aβ plaques. In addition, both studies agreed that there was a >60% reduction in Aβ plaque deposition in AD-Toxoplasma infected mice compared with uninfected mice [18,64]. However, it was unclear in these two studies whether this protection was conferred merely by the infection or if there were other factors involved since they used only one *Toxoplasma* strain (Type II) in their studies. To further study this limitation, Cabral et al. [65] focused on utilizing three *Toxoplasma* strains (Type I, II, and III) in their AD mouse model, thus reducing the influence of confounding factors. They found that infection with Type II *Toxoplasma* conferred better protection compared with Type I and III *Toxoplasma* strains. Additionally, there was a >60% reduction of Aβ plaque deposition, which was consistent with the two previous studies. Nevertheless, these authors also found similar TGF-β and IL-10 levels in the CNS of mice infected with Type II and Type III *Toxoplasma* strains, suggesting that elevated TGF-β and IL-10 levels may be not enough for cognitive protection. They also proposed a possibility consistent with Möhle’s study, by which Type II infection caused an accumulation of polarized macrophages/microglia in the brain that is effective in degrading Aβ plaques. In a recently in vitro study, a decrease in APP levels and, downregulation of PSEN2 and CSNK1A1 genes (both regulators of APP processing) following Type I *T. gondii* infection was found [66]. To our knowledge, the effects of Type II and Type III strains on APP processing have not been explored. 

**Figure 3:**
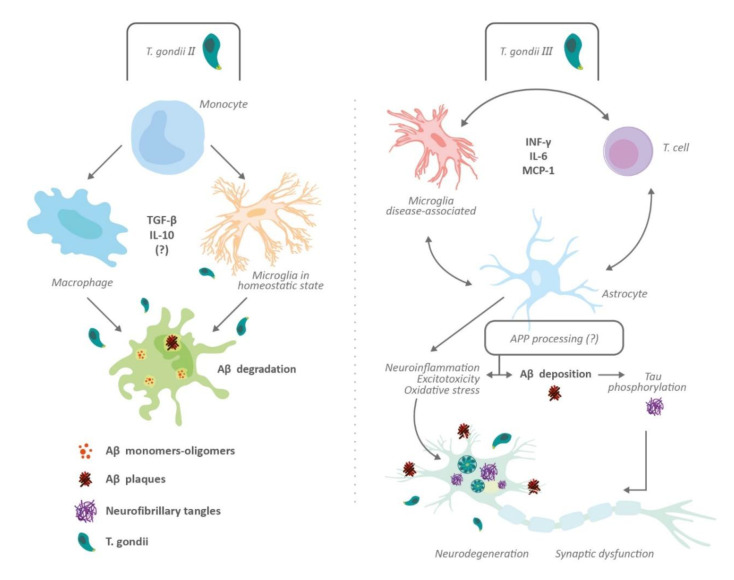
Impact of *Toxoplasma gondii* on amyloid processing and tau pathology. The effects of toxoplasmosis on Aβ plaque formation depends on *Toxoplasma* strains. Thus, Type II induces the activation of monocyte-derived cells (macrophage and microglia), possible immunomodulation and protective effects against Aβ deposition (left panel); Type III produces an elevated inflammatory response and nonprotective effects on Aβ deposition and tau phosphorylation (right panel). Neurodegeneration, synaptic dysfunction, and changes in neuronal microenvironment may underlie cognitive impairment induced by *T. gondii.* Type I *Toxoplasma* reduces amyloid precursor protein (APP) levels and induces downregulation of Presenilin 2 (PSEN2) and Casein Kinase 1 Alpha 1 (CSNK1A1) genes. The effects of Type II and Type III infection on APP processing is unknown. Abbreviations: Amyloid beta peptide (Aβ); amyloid precursor protein (APP); Casein Kinase 1 Alpha 1 (CSNK1A1); Interferon gamma (INF-γ); Interleukin (IL); Monocyte chemoattractant protein-1 (MCP-1); Presenilin 2 (PSEN2); Transforming growth factor-beta (TGF-β).

An additional factor related to toxoplasmosis and the pathogenesis of Aβ plaques is the presence of INF-γ [67]. This cytokine can produce opposite effects in the brain either by maintaining the latency of *T. gondii* or by promoting the expression of TNF and IL-1 by the microglia, leading to an enhancement of vascular permeability of T cells and NK cells [68]. Moreover, some human studies have associated IFN-γ with neurodegeneration. For instance, Meda et al. [69] reported synergism between IFN-γ and Aβ plaques in stimulating the production of reactive nitrogen products and TNF by microglia, generating cell injury. Likewise, Yamamoto et al. [70] showed synergism between IFN-γ and TNF when they are co-expressed in the brain, increasing Aβ production and reducing its clearance in an AD mouse model. Another study reported an association between IFN-γ and accumulation of Aβ_1-40_ and Aβ_1-42_ in astrocytoma cells, and human astrocytes isolated postmortem [71]. Moreover, the increased production of cytokines such as IL-1β and TNF owing to overstimulation of microglia may boost the activity and expression of secretases, contributing to Aβ deposition [15]. Furthermore, Browne et al. [72], through an AD mouse model, demonstrated that IFN-γ production by Aβ-specific Th1 cells induced microglia activation and deposition of Aβ plaques. This was ratified after using anti-IFN-γ antibodies, which weakened the response of Th1 cells on Aβ accumulation. Likewise, Mahmoudvand et al. [16] found that *T. gondii* could potentiate AD-like symptoms in Toxoplasma-positive mice after receiving a sub-dose of Aβ_1-42_, leading to memory and learning impairments similar to those seen in AD. However, other studies have shown a protective effect regarding IFN-γ over AD. For instance, IFN-γ overexpression showed a neuroprotective action in a transgenic mouse model. Those mice with overexpressed IFN-γ had a reduction of hyperphosphorylated tau compared with the control mice [68]. In the same way, selective blockade of programmed death-1 (PD-1) immune checkpoint, which induces IFN-γ-dependent activity, showed an improvement of memory in AD mouse models [73]. Finally, in a recent study [15], *T. gondii* infection in C57BL/6 mice induces major pathological hallmarks of AD, such as Aβ immunoreactivity and hyperphosphorylated tau protein, among others. Hence, there is a relationship between tau hyperphosphorylation, glutamate receptor dysfunction, neuroinflammation, and *T. gondii* (Figure 2 and Figure 3). 

In summary, cytokines and inflammatory reactions induced by *T. gondii* infection may have some anti-amyloid aggregation effects. However, it is still not clear if this may offer long-term benefits in cognitive function or neurodegeneration process observed in AD. Likewise, these apparently beneficial effects are depending on the specific *Toxoplasma* strain. More detailed studies are required to explore the synergistic actions of *Toxoplasma*, astrocytes, and microglia, over the development of neuroinflammation, amyloid plaques and related tauopathy.

## 4. Neurotransmitter Imbalance Induced by Toxoplasma

In addition to neuroinflammation, the relationship between *Toxoplasma* infection and cognitive dysfunction in AD might be explained by changes in several neurotransmitters involved in memory processing, including glutamate, GABA, and dopamine (Table 1). 

### 4.1. Glutamate

Glutamate is the main excitatory neurotransmitter in the CNS, and alterations in its function have been observed in AD. Several aspects of glutamate activity can be compromised in AD, including malfunction of ionotropic [85] and metabotropic receptors [86], together with altered astrocytic glutamate clearance [87]. Although no direct link has been established among *Toxoplasma gondii* infection, AD, and glutamate changes, some actions of this parasite on glutamatergic systems may help explain the appearance of the cognitive anomalies present in affected individuals.

The ionotropic glutamate receptor for N-methyl-D-aspartate (NMDAR) has been related to both AD and *T. gondii* infection. This receptor, which is present in various CNS cells, plays a crucial role in synaptic plasticity and cognition, including learning and memory processes [88]. Neuronal survival is compromised if insufficient synaptic NMDAR signaling is present, but excessive stimulation of glutamate is also neurotoxic, augmenting the risk for excitotoxicity. Both of these phenomena have been observed in AD, and it is likely that *T. gondii* infection may even induce them [85]. A recent study determined that the most affected subtype of NMDAR, after a *T. gondii* infection, is the NMDA 2D, which persists mainly in the hippocampal interneurons of adults [74]. Torres et al. [15] found AD signs in wild type mice after infection with *T. gondii*, demonstrated by loss of NMDAR signal, hyperphosphorylated tau and Aβ immunoreactivity in the brain, accompanied by alterations in olfactory sensitivity due to neuronal death in the olfactory bulb, memory impairment, and anxiety-like behavior. Furthermore, the continued presence of *T. gondii* tissue cysts in mice brains triggers the appearance of NMDAR autoantibodies, which decreases the availability of NMDA receptors via antibody-mediated receptor capping and internalization. It leads to behavioral abnormalities and synaptic loss [26]. In humans, acute infection with *T. gondii* has been reported to induce anti-NMDAR encephalitis [75]. This case report occurred in a nine-year-old child, who after two months, had a complete resolution of symptoms. It seems that the age of initial exposure to *T. gondii* may have a differential impact on the presence of autoantibodies and the downregulation of NMDAR subunits, which could be critical for the determination of several neurobehavioral abnormalities, such as dementia, seizures or schizophrenia [77]. 

In addition to NMDAR, other glutamate-related proteins have also been shown to be downregulated in *T. gondii* infection, including the fast-acting glutamate ionotropic receptor alpha-amino-3-hydroxyl-5-methyl-4-isoxazole-propionate (AMPA) (subunit GluA2), SH3 and multiple ankyrin repeat domains 3 (Shank3) and excitatory amino acid transporter 2 (EAAT2; also referred to as GLT-1) [26]. GluA1 and GluA2 subunits are essential for the regulation of intracellular sorting and degradation of the AMPA receptor, necessary to maintain homeostatic levels of this glutamate receptor [89]. Most AMPA receptors within the hippocampus and cerebral neocortex contain the GluA2 subunit [90,91]; furthermore, the function of the AMPA GluA2 subunit has been shown to be compromised by the presence of Aβ in AD [92]. Moreover, the GluA2 subunit determines calcium entry to the cell through the AMPA receptor [93]. If GluA2 is present within the AMPA receptor, calcium permeability is markedly reduced, while the presence of GluA1, GluA3, and GluA4 subunits make the AMPA receptor highly permeable to calcium [94]. Therefore, the downregulation of GluA2, as seen in *T. gondii* infection, may alter intracellular calcium dynamics, even placing the cell at risk of excitotoxicity. Shank3 is a large scaffold postsynaptic density protein implicated in the formation of both dendritic spines and synapses, and it also plays a role in the regulation of the expression of metabotropic glutamate receptor 5 (mGluR5) [95]. Downregulation of this protein has been shown to reduce spine density in hippocampal neurons [96], and recently, it has been reported to be significantly downregulated in sporadic AD [97]. Further research involving Shank3, AD, and *T. gondii* will be useful to determine a relationship between these factors. EAAT2 is present almost exclusively in astrocytes and is believed to be the primary transporter responsible for glutamate clearance in the brain [98]. Abnormal function of EAAT2 can disrupt the glutamate balance in the synaptic cleft and alter the function of postsynaptic neurons, increasing the probability of hyperexcitability, excitotoxicity, and cell death [99]. Inhibition of glutamate transport and malfunction of EAAT2 have been observed in AD, explaining in part some of the synaptic alterations observed in this disease [100,101]. Mice infected with *T. gondii* presented increased extracellular levels of glutamate due to disrupted astrocytic regulation, which resulted in neuronal pathology and loss of electroencephalographic (EEG) power [76]. The same paper reported that neuronal function was rescued by the upregulation of EAAT2 following the administration of the beta-lactam antibiotic ceftriaxone. Another study performed in mice also observed marked downregulation of EAAT2 in synaptosomes in the cortex and hippocampus (but not in the thalamus or striatum), and this downregulation was also rescued with the use of the sulfonamide antibiotic sulfadiazine [26]. The researchers of this investigation suggest that a more global impairment of glutamatergic synapse function is related to *T. gondii*-induced neuroinflammation. Augmented neuroinflammation is also one of the most representative findings in AD, impairing cellular repair processes and facilitating the maintenance of pathological changes [102]. These observations provide a plausible connection between EAAT2 glutamate disruption and neurobehavioral changes, similar to those present in dementia, induced by *T. gondii* infection.

### 4.2. GABA

Gamma-aminobutyric acid (GABA) is the main inhibitory neurotransmitter in the human brain. Although GABAergic systems have been much less studied than cholinergic or glutamatergic system in AD, some evidence has arisen that indicates alterations in GABA in this disease [103]. Temporal cortices from human brains with AD show a functional remodeling of GABAergic neurotransmission, with a reduction in GABA currents, together with a decrease in mRNA expression and protein synthesis of the main GABA receptor subunits α1 and γ1 [104]. Furthermore, Aβ has been observed to weaken synaptic inhibition through the downregulation of GABA_A_ receptors in Wistar rats [105]. GABA is not limited to inhibitory neurotransmission but has also been involved in cellular migration and metastasis in the body [106,107]. In fact, *T. gondii* infection induces hypermigration of infected dendritic cells through GABAergic signaling; therefore, the GABAergic system can be exploited by an intracellular pathogen to modify the motility of the host cell and to potentiate systemic dissemination [36]. A similar strategy has been reported in the CNS, where *T. gondii* activates hypermigration of microglial cells, but not astrocytes, via GABAergic signaling in order to facilitate parasite dispersion in the brain parenchyma [78]. This so-called Trojan horse mechanism of hijacking GABAergic signaling appears to involve the activation of GABA_A_ receptors and L-type voltage-dependent calcium channels [78,79]. However, a study performed on mice chronically infected with *T. gondii* showed no effect in the infection status or interaction in either the cortical or subcortical areas (neocortex, thalamus, striatum) regarding GABA_A_ α1 [26]. Nonproliferating *T. gondii* tachyzoites have been reported to both synthesize GABA and utilize exogenous GABA [108]. Although GABA is not actively secreted by tachyzoites, it is possible that GABA liberation from dead bradyzoites could alter the GABAergic balance and produce neurobehavioral changes in infected individuals. In addition, *T. gondii* infection appears to alter the inhibitory function of GABAergic signaling. Mice infected with *T. gondii* presented changes in the distribution of glutamic acid decarboxylase 67 (GAD67), an enzyme that catalyzes GABA synthesis in the brain, facilitating the development of seizures [80]. Likewise, expression of frontal cortex GAD67 was reduced in juvenile and adult mice infected with *T. gondii* [77]. GABA hypofunction can also be related to glutamate, as GABAergic interneurons express NMDAR; therefore, dysfunction of these NMDARs could decrease GABAergic activity and consequently diminish neuronal inhibitory control [74]. Whether changes in GABA due to *T. gondii* infection may be related to the appearance of pathological changes similar to those of AD is currently unknown, although GABAergic function seems to be compromised in both conditions.

### 4.3. Dopamine

Dopamine is involved in functions regarding movement, memory, motivational behavior, and mood in humans [109]. Due to its multiple functions, dopamine dysregulation may be related to the onset of neuropsychiatric disorders, including AD. Actually, in a recent network meta-analysis, it was found a reduction in dopamine receptor levels in patients with AD [110]. Several studies have shown a relationship between dysfunction of the dopaminergic system and the development of AD, specifically in the ventral tegmental area (VTA), the nucleus accumbens (NAc), and the locus coeruleus (LC). For instance, Nobili et al. [111] showed that the degeneration of dopaminergic neurons in the VTA plays a major role in the memory deficit and cognitive impairment seen in mice with the APPswe mutation (an animal model of AD) due to a loss of inputs from subcortical structures to the hippocampus. Moreover, a Tg2576 mouse model of AD demonstrated that degeneration of the VTA impairs memory and cognitive functions not only through a diminished direct input, but also due to a dysfunction of the hippocampus-NAc connections [112]. Hence, there is robust evidence that suggests that an imbalance in dopaminergic systems contributes to the development of AD.

Toxoplasma-induced dopamine dysfunction has also been studied in order to shed some light on the relationship between the parasite’s infection of the CNS and AD development [113]. For instance, *T. gondii* genome has two genes, aromatic amino acid hydroxylase 1 and 2 (AAH1 and AAH2) that code for tyrosine hydroxylase (the enzyme responsible for the production of L-DOPA) and *T. gondii* infection decreased expression of Dopamine Transporter (DAT) and Vesicular Monoamine Transporter 2 [81]. These effects, may increase the dopamine availability in the synaptic cleft. Moreover, in a study conducted by Ting Wang et al. [50] in mice infected with *T. gondii*, an evident change was observed in the dopaminergic pathways caused by a significant reduction in *DRD1*, *DRD2*, *DRD4*, and *GRK6* gene expression, thus reducing dopamine receptor availability and rise dopamine concentrations. Supporting these findings, in vivo and in vitro studies have shown increases in dopamine synthesis and release from dopaminergic cells and in the brain tissue of mice infected with *T. gondii* [82,83]. This increase in dopamine levels can affect some of the brain cortex functions, including executive, motor, memory, and emotional operations or induce psychiatric symptoms associated with neurodegenerative disorders. It can be explained because the cortex is primarily controlled by subcortical projections, and one of these comes from the dopaminergic system mainly regulated by dopamine D1-like receptors also involved in cognitive function, synaptic function, and neuroprotection [114]. Some authors have reported that the hyperfunction of dopamine neurotransmission leads to dysregulation of prefrontal cortex functions, causing cognitive impairment [115]. In addition, the accumulation of intracellular dopamine leads to cell damage caused by improper packaging of dopamine into vesicles. It contributes to the production of free radicals, which cause dendritic spine damage, a pathological finding already described in toxoplasmosis [116]. Furthermore, other molecular pathways involved in dopaminergic dysfunction have been studied. Jianchun Xiao et al. [84] found a significant increase in the transcription of MiR-132, a noncoding RNA sequence (key in the regulation of neural development), in human neuroepithelioma cells infected with *Toxoplasma*. Said upregulation translated into a decrease in the dopamine D1 receptor family (DRD1 and DRD5), the MAO-A enzyme, and dopamine-mediated transduction proteins such as DARPP-32, all of which led to an overt imbalance in dopaminergic pathways and accumulation of intermediate metabolites. In another study conducted by Syn et al. [66], an epigenetic alteration within the methylome of human WERI-Rb-1 eye cell line infected with *Toxoplasma* Type I resulted in perturbed DARPP-32 feedback in cAMP dopaminergic signaling. These changes may lead to a disruption of synapse structure, neuro-progenitor cell proliferation, axonal guidance, and neuronal migration in the developing brain. 

On the other hand, different studies have found a reduction in dopamine concentration or even an innocuous response to infection. For instance, Goodwin et al. [117] did not find changes in dopamine levels in congenitally *T. gondii* infected mice compared to controls. However, their mice expressed behavioral changes, which suggest that another transmitter system could be implicated in these findings. 

## 5. Toxoplasmosis and ApoE

ApoE is the main cholesterol carrier involved in axonal development, metabolic function, regulation of Aβ aggregation, and injury repair in the brain [118]. One of its functions is to redistribute lipids derived from neurodegeneration to other cells that require lipids for proliferation, remyelination, or membrane repair [119]. The presence of ApoE polymorphic alleles (ε2, ε3, and ε4) have been considered genetic factors for AD development. For instance, ApoE ε4 carriers have a higher risk of developing sporadic AD compared with those carrying ApoE ε3, while ApoE ε2 carriers are thought to have a decreased risk [118]. The risk of having AD given by ApoE ε4 is due to an increase in pro-inflammatory processes and a decrease in anti-inflammatory ones. For example, ApoE ε4 is related to arginine increment by microglia, increasing reactive oxygen species [120]. To further elucidate such findings, Lin et al. [121] studied the molecular differences in an ApoE ε3 and ApoE ε4 in a pluripotent stem cell-induced neuron, astrocyte, and microglia model. In their study, ApoE ε4 neurons exhibited an increase in synaptic activity, which correlated with increased Aβ_1-42_ production compared with ApoE ε3 neurons. Additionally, astrocytes expressing ApoE ε4 were shown to decrease ApoE production compared with ApoE ε3-expressing astrocytes; this finding is related to an increase in cholesterol biosynthesis from ApoE ε4 astrocytes, which suggests that the ApoE ε4 allele hinders astrocyte lipid metabolism. Likewise, ApoE ε4 astrocytes displayed decreased lysosome-independent and dependent Aβ_1-42_ uptake and clearance, thus supporting the contribution of the ApoE ε4 allele to Aβ_1-42_ accumulation and shedding light upon its role in AD’s pathophysiology. Finally, in ApoE ε4-expressing microglia, besides from interestingly acquiring an abnormal morphology with fewer and shorter membrane processes, the authors found an upregulation of immune response genes as well as an altered Aβ_1-42_ uptake when compared with ApoE ε3-expressing microglia, findings suggestive of a pro-inflammatory state response. Lastly, in a recent case report from Arboleda-Velasquez et al. [122], a patient carrying a Presenilin 1 mutation did not experience cognitive decline until her eighth decade of life, three decades after the expected onset of cognitive decline; additionally, the patient had two copies of the ApoE ε3 Christchurch (R136S) mutation. Such findings are compatible with the above-mentioned role of ApoE ε3 as a protective factor against AD development. 

In toxoplasmosis infection, the parasite affects the cholesterol from host neuron cells, potentiating the cognitive impairment. One research group has studied the association of ApoE genotypes on dementia in patients with latent toxoplasmosis. Yahya et al. [123] found in a cohort from Egypt that regardless of ApoE ε4 carriage, *Toxoplasma* patients have a higher risk of developing dementia. The predominant allele found in that cohort was ApoE ε3, followed by ApoE ε4 and ApoE ε2 alleles, which was consistent with previous studies that suggested the most common allele in Egypt is ε3. Despite this finding, much more research is needed to investigate whether *T. gondii* affects ApoE function or not. Besides, it should be explored if different strains of *Toxoplasma* may produce differential effects regarding the ApoE alleles.

## 6. Final Considerations and Conclusions

*T. gondii* infection induces functional changes in many areas of the CNS, including those involved in essential brain activities such as memory, executive functions, behavior, and motor responses, which are also compromised in AD. Several CNS cell types, including astrocytes, microglia, and neurons, are affected by *T. gondii*, altering the physiological action of gliotransmitters and neurotransmitters. Three main neurotransmitters, glutamate, GABA, and dopamine, have been reported to be affected by *Toxoplasma*. Glutamate activity seems to be altered, due to the presence of autoantibodies against NMDA, downregulation of AMPA receptors, and disruption of astrocytic EAAT2 transporters. The parasite uses a Trojan horse mechanism through GABA signaling, facilitating dispersion in the brain parenchyma. Furthermore, the presence of *T. gondii* affects dopaminergic activity, which may alter cognitive, behavioral, and motor activities. Similar neurotransmitter changes have also been observed in AD; thus, a connection between the parasite infection and dementia is plausible. Despite these observations, more experimental research is needed in order to explore and clarify the role that *T. gondii* plays in neurotransmission changes described in AD. 

From a clinical standpoint, there is a controversial relationship between toxoplasmosis and dementia; however, some reports indicate that infection by *T. gondii* could be considered a risk factor for AD or exacerbate the cognitive impairment caused by other types of neurological disorders. *Toxoplasma* may affect amyloid processing, leading to Aβ immunoreactivity, hyperphosphorylated tau protein, and loss of NMDA receptors. In addition, this parasite stimulates the immune response in the CNS, affects the local microenvironment, and activates NF-κB signaling in astrocytes generating pro-inflammatory cytokines and chemokines, excitotoxicity and oxidative stress. In contrast, the chronic low-grade neuroinflammation induced by toxoplasmosis may have protective effects on the dissemination of *T. gondii* through the CNS and it may reduce Aβ plaque and hyperphosphorylated tau formation. However, this may be due to the specific strain of *Toxoplasma*, as type II may offer some protection, while type I and III are harmful. 

Interaction between immune cells such as monocyte-derived macrophages, microglia and T lymphocytes and specific *T. gondii* strains may lead to changes in amyloid processing and neuronal microenvironment. This relationship is a key factor in the understanding of the pathophysiology of cognitive impairment in several neuropsychiatric and neurodegenerative conditions associated with this parasite. Therefore, we suggest the conduction of new studies regarding the role of *T. gondii* in APP processing, beta and gamma secretase function, tau hyperphosphorilation and interaction with amyloid degrading enzymes such as Neprilysin (NEP) and Insulin-Degrading Enzyme (IDE). Further studies are needed to explore the effects of chronic toxoplasmosis over cognitive impairment, including AD, in individuals with specific genetic markers (i.e., APOE-ε4) and environmental risk factors. 

## Figures and Tables

**Table 1:** Summary of the *Toxoplasma gondii* effects on main neurotransmitter systems.

Neurotransmitter	Toxoplasma Effects	Study Type	References
Glutamate	Cross-reactivity with NMDA-2D receptors.	In silico (UniProt database and Peptide Match program)	[74]
	AD signs associated with loss of NMDAR expression and neuronal death.	In vivo and in vitro (C57BL/6 mice).	[15]
	Downregulation of synaptosomal EAAT2, AMPA receptor subunit GluA1, and the NMDA receptor subunit GluN1	In vitro (Naval Medical Research Institute—NMRI-mice)	[26]
	Development of anti-NMDA encephalitis.	Case report	[75]
	Reduction in the astrocytic glutamate transporter, GLT-1 and increase in extracellular levels of glutamate. Abnormal EEG recordings.	In vivo and in vitro (C57BL/6 and BALB/c mice)	[76]
	Elevation of GLUN2 autoantibodies and reduction in Glun2A expression (NMDAR subunits). Reduction in the vesicular glutamate 1 transporter (VGLUT1) and post-synaptic density 95 (PSD-95).	In vitro (BALB/c mice)	[77]
GABA	Dendritic cells hypermigration through GABAergic signaling which allows parasitic systemic dissemination.	In vivo and in vitro (C57BL/6 mice bone marrow-derived DC and human monocyte-derived DC)	[36]
	Increased microglial cells hypermigration via GABAergic transmission.	In vitro (C57BL/6 mice astrocyte and microglia cell cultures)	[78]
	Activation of GABA-A receptors and L-type voltage-dependent calcium channels to modulate microglial activation and migration.	In vivo, ex vivo and in vitro (cell line NE-4C, mouse bone marrow-derived DCs and C57BL/6 mice)	[79]
	Diminished expression and altered cortical GAD67 distribution. Reduction in GABAergic transmission.	In vivo and in vitro (BALB/c and C57BL/6 mice)	[77,80]
Dopamine	Reduction in DRD1, DRD2, DRD4, and GRK6 gene expression, reducing receptor availability and increasing dopamine concentration.	In vitro (BALB/c mice)	[50]
	Decreased expression of Dopamine Transporter (DAT) and Vesicular Monoamine Transporter 2. Increasing locomotor activity to dopamine psychostimulants.	In vivo and in vitro (BALB/c mice)	[81]
	Increased dopamine synthesis and release. Increased DOPA decarboxylase (DDC) levels.	In vivo and in vitro (rat pheochromocytoma PC12 cells and Swiss Webster mouse)	[82,83]
	Decrease in D1-like receptors (DRD1, DRD5), MAO-A, and DARPP-32 gene expression, via MiR-132 RNA transcription.	In vitro (human neuroepithelioma cell line and CD-1 mice)	[84]
	Disruptions of dopamine-related pathways with DARPP-32 feedback and APP production.	In vitro (human WERI-Rb-1 eye cell line culture). Genome-wide analysis	[66]
